# Sea ice records over more than a century at an observatory facing the Okhotsk coast of Hokkaido, Japan

**DOI:** 10.1038/s41597-025-05277-1

**Published:** 2025-06-13

**Authors:** Takahiro Toyoda, Kento Tsukagawa, Yusuke Kimura, Honoka Abe, Meiji Honda, Yuki Yamada, Miku Amano, Yoshihiro Tachibana, Yuka Sawa, Keisuke Morimoto, Hiroaki Ueda, Katsushi Iwamoto, Kimihiro Itoh, Fumitake Shido, Hitoshi Tada, Hideki Kaneko, Katsuya Toyama, Naohiro Kosugi, Yusuke Ushijima, Masao Ishii

**Affiliations:** 1 Meteorological Research Institute, Japan Meteorological Agency (JMA), Tsukuba, Japan; 2 Abashiri Local Meteorological Office, JMA, Abashiri, Japan; 3 Graduate School of Science and Technology, Niigata University, Niigata, Japan; 4 Faculty of Science, Niigata University, Niigata, Japan; 5 Weather and Climate Dynamics Division, Mie University, Tsu, Japan; 6 Graduate School of Science and Technology, University of Tsukuba, Tsukuba, Japan; 7 Faculty of Life and Environmental Sciences, University of Tsukuba, Tsukuba, Japan; 8 City of Mombetsu, Mombetsu, Japan; 9 Sapporo Regional Headquarters, JMA, Sapporo, Japan; 10 Wakkanai Local Meteorological Office, JMA, Wakkanai, Japan; 11 Fukui Local Meteorological Office, JMA, Fukui, Japan; 12 Center for Marine Environmental Studies, Ehime University, Matsuyama, Japan

**Keywords:** Physical oceanography, Physical oceanography

## Abstract

Socio-economical activities around the Okhotsk coast of Hokkaido, Japan are strongly influenced by local sea ice variations, which are determined by larger-scale atmospheric drivers in the North Pacific. Here, we constructed the long-term sea ice observational data from the 1890s by combining data from the 1940s, already released by the Japan Meteorological Agency prior to this study, with further data shared for the first time in this study. For this purpose, old hand-written reports and sketches stored in the Abashiri Local Meteorological Office were investigated. For each year, first and last sea ice dates and daily sea ice variations recorded visually by sighting the ice were digitized to provide continuous time series to the present. In addition, we introduced a new reconstruction approach from satellite data for the date of last annual drift ice occurrence, for which observations are lacking after 2021. The data presented here are of value in understanding the long-term climate variability in the Sea of Okhotsk and the North Pacific.

## Background & Summary

Long-term observational records that capture specific aspects of the climate system have provided scientific foundations for understanding climate variability and change^1–3^. Observation data for the oceanic fields are generally scarce compared to those for the atmospheric fields due to difficulties in the measurements^4,5^. Although remote sensing has greatly extended the areas of observations since the 1970s, data before the satellite era are limited, for which efforts of archiving previous data have been continued^6^. Records of sea ice variations before the satellite era^3,7^ are particularly limited, which restricts our knowledge of the long-term climate variability associated with the sea ice and also contributes to the large uncertainty of reconstructions and predictions of polar regions’ climate in modeling studies^8,9^.

Japan Meteorological Agency (JMA) has kept the long-term atmospheric and oceanic observations, such as on the repeat hydrographic section along 137°E since 1967^10^. In terms of the sea ice, the observatories of the JMA along the Okhotsk coast of Hokkaido, Japan have conducted direct observations of sea ice at the observatory sites. Part of these data (e.g. from 1956) are available from the web site of the JMA (see below). Among the observatories, Abashiri is a key location, downstream of sea ice drift along the Eastern Kamchatka Current from the north; interannual variations of the sea ice amount along the Okhotsk coast of Hokkaido are mainly captured by those at Abashiri. Further, statistical analyses suggested that the interannual variations of the sea ice there (first and last dates and accumulated sea ice amount of the year) are attributable to the North Pacific climate conditions (e.g. strength of the Aleutian Low)^11,12^. In addition, other studies indicated that the variations in the ocean-sea ice fields in the Sea of Okhotsk can impact the basin-scale atmospheric field through processes such as teleconnections and cold air advections and also the oceanic intermediate water in the North Pacific through dense water formation^13–17^. These interactive features between the North Pacific and the Sea of Okhotsk suggest that the time series of the above sea ice observations can serve as a proxy or indicator of North Pacific climate variations not captured by other widely-used climate indices (e.g. North Pacific Index^18^). Note that previous studies^11,12^ used the time series from 1956 as already published by the JMA. However, earlier data extending back to the 1890s had been summarized in the literature^19^; thus, the data before the Central Meteorological Observatory was changed to the JMA in 1956 exist but have not been released publicly to date.

**Fig. 1:**
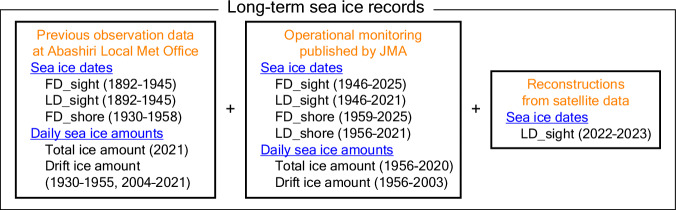
Schematic overview. The long-term observational sea ice records for sea ice dates and daily sea ice amounts as provided in this study. See text for details of the variables.

Here, we investigated the sea ice records, which are mostly handwritten, stored in the library and seismographic room of the Abashiri Local Meteorological Office. The purpose of this study is to rescue and digitize these relevant records and provide the continuous time series along with those already available from the JMA web site. Because part of the operational daily observations at Abashiri were ended in 2021 with other observatories ceasing even earlier, we also attempted to extend the time series (toward the present) by using the relationship between the existing time series and satellite data. Thus, unprecedented long-term observational sea ice records over more than a century (Fig. 1) are provided for the Sea of Okhotsk in this study, which may be of use in studies of the North Pacific climate along with previous long-term sea ice records in the Bering Sea^3,7^.

## Methods

### Operational monitoring by the JMA

**Fig. 2:**
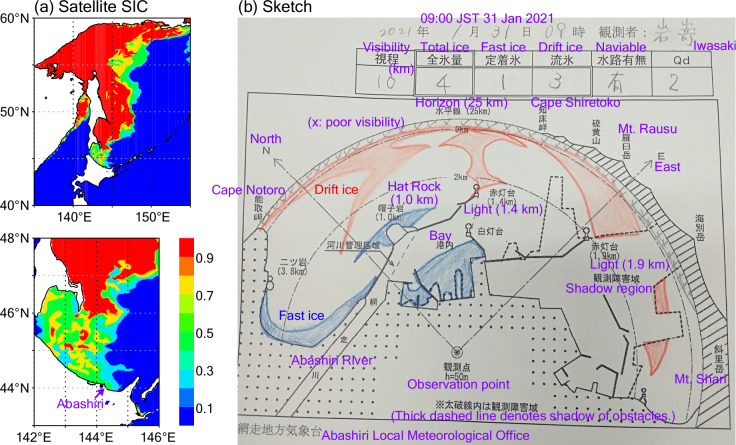
An example of operational monitoring. (**a**) Distribution of sea ice concentration (SIC) based on satellite observations on 31 January 2021. Operational daily SIC estimates with resolution of about 2 km^23^ are used. (**b**) Sketch of the operational sea ice monitoring from the Abashiri Local Meteorological Office on the same day by Mr. Iwasaki. Land-fast ice and drift ice are indicated by blue and red shading, respectively. Complemental descriptions in English (purple font) are added corresponding to original descriptions in Japanese (black). FD_shore in 2021 was specified on this day.

First, we describe the recent observations operationally conducted and published by the JMA (https://www.data.jma.go.jp/gmd/kaiyou/db/seaice/hokkaido/hokkaido_static.html). The operational officer monitors the sea ice amount (fractional sea ice distribution) within the visibility of about 20 km from the Abashiri Local Meteorological Office building (44.02°N, 144.28°E) of about 50 m height above sea level. The observation time was previously 10:00 JST (UTC + 09:00) every day during the sea ice season^20^, but was changed to 09:00 JST (in 2004) due to the daily report requirement until 09:20 JST^21^; additional observations (not at a fixed time) are sometimes conducted for determining the special dates of sea ice arrival/retreat (see below) or when the regular observations are not possible according to weather conditions. Total sea ice consists of drift ice and fast (land-fast) ice. These ice types are regularly distinguishable based on the location and appearance (e.g., reflectivity), since fast ice generally forms in the bay and along the coast affected by land-oriented fresh water (e.g., from the Abashiri River) and is thinner and with less snow on it than drift ice. It is noted in the handbook^21^ that ice that forms in the bay and is artificially drained out of the bay is counted as fast ice (the amount would be tiny); and also that drift ice that remains attached to the coast for a period of time without moving is counted as drift ice (although it is land-fast ice by general definition, the ice amount measurements emphasize the orientation: fast ice is formed by growth from the coastline)^20^. The sea ice areas for these types are sketched separately (Fig. 2) and are reported as 10ths of the observation area covered by ice (quantile): e.g. 0 for no ice and 10 for full ice cover; additionally, “0+” for very little fractional ice (slightly greater than 0 but less than 1) and “10−” for almost full ice cover with small gaps (greater than 9 but slightly less than 10) are used (after 1951) (Canadian Ice Service uses similar classifications, e.g., “9+” for concentration quantile between 9 and 10^22^). When all ocean surface is visible, the bay area (inside of jetty lines and the line between the red (1.4 km) and white lights is accounted for 11%; the area outside of the bay and within the 2 km circle is accounted for 30%; the area between the 2 km and 9 km circles is accounted for 48%; and the area out of the 9 km circle is accounted for 11% (these area fractions have historically been modified according to the changes in the jetty lines and shadow region, for example. When the visibility is not full, the fractional area is calculated within the visibility. When the visibility is less than 5 km, the ice amounts are determined as unknown. In addition to the daily monitoring, four special sea ice dates are defined for each year: first date of drift ice in sight (“FD_sight”, hereafter), first date when waters are not navigable by ships as drift ice connects to land-fast ice (first date of drift ice on shore; “FD_shore”), last day (of several days) when waters become navigable as ice retreats to a fraction less than 5 (first date of shore lead appearance; “LD_shore”), and last date of drift ice in sight (“LD_sight”). Duration of drift ice is calculated from FD_sight and LD_sight. Presently, only FD_sight and FD_shore are monitored. Thus, the sea ice dates at the Abashiri Local Meteorological Office as published from the JMA are (1) FD_sight from 1946 to 2025, (2) FD_shore from 1959 to 2025, (3) LD_shore from 1956 to 2021, and (4) LD_sight from 1946 to 2021. For the daily sea ice amounts, the JMA web site describes the total sea ice amount during 1956–2021 and the drift ice amount during 1956–2003. In this study, as a quality control for the period 1956–2003, the drift ice amount was reduced to the total ice amount when the former is larger than the latter (the changes are no larger than 1; e.g. from 10 to 10−). In addition, based on a simple check (consistency among data of the daily report), the symbol on 18 February 1975 was corrected to “−” (from “+”). See Technical Validation section for statistical validations. The source of these data is referred to as “JMA” in this study.

### Previous observations

Based on our investigation, sea ice observation records from 1892 remain at least partly in the library and seismographic room of the Abashiri Local Meteorological Office. Particularly, *Sea Ice Accumulative-Year Original Ledger* (see Table 1 for the original Japanese names of this document and other Japanese references) summarizes the sea ice observation results in the old years (e.g., FD_sight from 1892 to 1998; daily drift ice amount from 1930 to 1983). In addition, daily sketches can be referred to as bases of the values in the ledger for several years. The older version of the ledger which was used until 1956 remains as a copy; another version contains the data until 1994. In this study, we digitized and performed quality checks on the data in these ledgers to create a continuous time series of sea ice observations from 1892 to 2025. Note that it has been discussed that the JMA cannot treat the observations before 1945 as official data since these have not been published as a printed document of the JMA and also that differences in the observation method and the thresholds for the sea ice dates between before 1945 and after 1946 might affect the homogeneity of the records. In particular, definition of FD_shore has been changed several times: the first date when the drift ice amount becomes 10 or 10− (previously): the first date when the drift ice with the amount $\ge$8 connects to the shore or land-fast ice^20^ (this change was probably made around 1959; at least before 1966); the first date when waters are not navigable by ships as drift ice ($\ge$7) reaches approximately 80% of the coastline (or fast ice rim) within sight, blocking shipping routes (after around 1984); and the operational FD_shore as described in the previous subsection (after 2004). For definition of LD_shore, the condition that waters are navigable was added in 2015^21^ to the ice amount ($\le$5) condition^20^. Definitions for FD_sight and LD_sight have not been changed basically. We investigate the statistical features of the data to assess the possibility of constructing long-term records toward the present (in Technical Validation section). The source of these data stored in the Abashiri Local Meteorological Office is referred to as “ABA” in this study.

**Table 1:** Original names of documents in Japanese whose provisional English names are referred to in the main text.

Provisional English name	Japanese name
*Sea Ice Accumulative-Year Original Ledger*	海氷累年原簿
*Sea Ice Observation guidelines*^20^	海氷観測指針
*Sea Ice Operations Handbook*^21^	海氷業務便覧

### Recent observations

Although the official release of the drift ice amount ended in 2003, the sketches were continuously executed till 2021. We have constructed the daily drift ice amounts during 2004–2021 based on the sketches. The source of these recent data is also referred to as “ABA” in this study. Unfortunately, sketches were not made frequently during 2004–2007 (15, 25, 15, and 17 sketches, respectively), we used both available drift ice data (from sketches) and total ice data (fully available except for bad weather days; JMA); specifically, a constant difference between total and drift ice amounts (average of values for the previous and next days of the gap) is assumed during each gap period and the drift ice amounts are calculated by subtracting the constant from the total ice amounts (set to 0 if the result falls below 0). For the gap periods in the other years (a few days at most), linear interpolation was used as for the official JMA data^21^. The sketches were also used for providing the total ice data for 2021 since the data have not yet been released by the JMA. In addition, the sketches were used for modifying some of the JMA total ice amounts. This is because the JMA data are based on the daily morning reports but additional observations were sometimes conducted afterward when the regular observation (at 9:00 JST) could not determine the ice amounts due to poor visibility (<5 km).

### Recent reconstructions

**Fig. 3:**
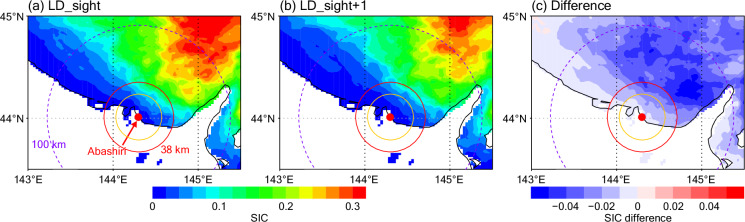
Satellite SIC distributions. (**a**) Composite of LD_sight days. (**b**) Composite of the days after LD_sight (LD_sight + 1). (**c**) Difference between the composites (LD_sight + 1 minus LD_sight). Yellow, red, purple circles denote 25, 38, and 100 km ranges, respectively, from the Abashiri Local Meteorological Office (red closed circle) in each figure.

**Fig. 4:**
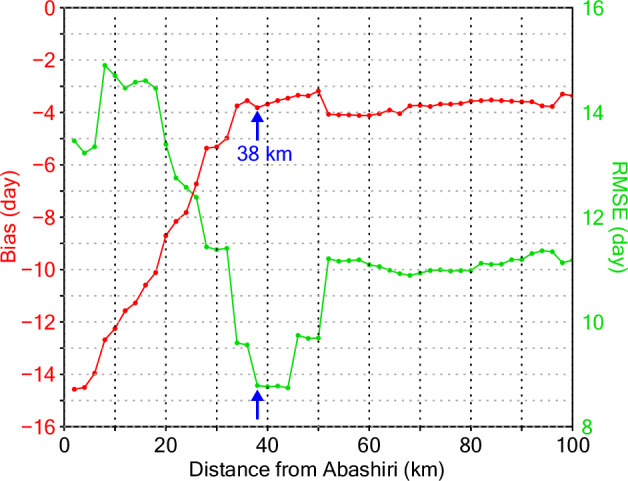
Bias and RMSE. Biases (red; left axis) and root mean square errors (RMSEs; green; right axis) of the LD_sight estimations as functions of the range distance as applied to the satellite SIC data. Values at the distance of 38 km are indicated by blue arrows (see text).

While operational monitoring of FD_sight has been continued, monitoring of LD_sight was ended in 2021, since the commercial requirements are higher for the former. In this study, we attempted to reconstruct recent time series of LD_sight by using the relationship between the existing time series and the satellite SIC (sea ice concentration) data^23^, whose overlapping period is 1978–2021. The SIC data are derived from visible, infrared, and microwave images from satellites and visual reports of aircrafts of the Japan Maritime/Ground Self-Defense Force and the Japan Coast Guard through a mix of methods that involve human analysis, with relatively high resolution of 0.05° covering the Sea of Okhotsk (see the previous study^24^ for more details). The SIC distributions for the composites of the LD_sight and the day after LD_sight (LD_sight + 1) actually show SIC decreases around Abashiri (Fig. 3). In details, the large reduction area distributes to the northeast of Abashiri with relatively small signals along the coast. These features are consistent with the correlation map between LD_sight and April SIC distribution as shown in the previous study^11^ (their Fig. 6b). In this study, we focus on the difference of the estimated LD_sight with different distances from Abashiri. Note that the distance of the horizon from the observation location is about 25 km (yellow circle). On the other hand, we can suppose that the satellite data provide smoothed (interpolated) distributions based on limited data sources on this local scale. Hence, timing of the SIC disappearance within the 25-km range in the satellite data might not reflect the observed LD_sight. In addition, previous study suggested that the LD_sight is affected by the large-scale surface wind anomalies associated with the Aleutian Low variations^11^. Thus, we tested several distances (2–100 km; 2 km intervals) for the range where mean SIC value is calculated (SICs are averaged over the region within the range and between the east and the north; e.g. Fig. 2b). For each tested distance, the mean SIC values on LD_sight and LD_sight + 1 were compared during 1978–2021 and the mean value (i.e., $({{SIC}}_{{LD\_sight}}+{{SIC}}_{{LD\_sight}+1})/2$; averaged over the above years) was used as the threshold to estimate the LD_sight from the satellite SIC data. Bias and root mean square error (RMSE) of the estimated dates from the observed dates are presented in Fig. 4 as function of the distance. Negative biases are generally seen, i.e. the estimated dates are too early; the magnitude of the bias decreases with the distance increased until about 40 km. In addition, the RMSE also decreases broadly, although step-like variations are seen possibly due to the representative resolution of the SIC data. It can also be considered that a value close to an integer number is appropriate for the bias correction since LD_sight of integer is useful. Hence, we chose 38 km as the range representing/reflecting the local SIC variations in the satellite data, whose bias and RMSE values are −3.8 and 8.8 days, respectively. Although the RMSE value is not so small, this is still larger than the interannual variability of LD_sight (15.6 days), supporting our approach. Thus, the distance 38 km (threshold SIC value of 0.067) was used for reconstructing the LD_sight during 2022–2023 (bias corrected). Durations of drift ice (from FD_sight to LD_sight) are also calculated for these years. Although these are reconstructions for only two years (SIC data in 2024 have not yet been available), this approach would be useful for future estimations of this index. The source of these estimations is referred to as “EST” in the data records. As for another index LD_shore which has not been monitored recently, it would be determined so locally that reconstruction from the broad-scale data as above would be difficult.

## Data Records

The data have been deposited on figshare^25^.

We have merged the time series of the observational sea ice amounts and dates at Abashiri as described above. For the sea ice dates, we combined the ABA data during 1892–1958 and the JMA data during 1959–2025, since FD_shore values during 1946–1958 are not defined in the JMA data. Note that the other values during the overlapping period (1946–2015) are consistent between the two datasets. LD_sight values during 2022–2023 adopted the reconstructed estimates. These time series are provided via a repository as ‘Si_date_1892–2025.txt’^25^, which contains yearly values in an ascii format and comments on the data source (see the header part for the detailed format to read the data).

For the daily total ice amounts, the JMA data during 1956–2021 were rearranged (with small modifications due to quality controls) as ‘Total_ice_1956–2021.txt’^25^. For each year, the data part contains six monthly lines with daily values (in a csv format) from previous December to May (e.g. from December 2000 to May 2001 in the block for 2001). For the daily data (with one character for each day), 0–9 denote the area fraction in 10th quantile; “*” denotes full ice cover (10); “+” and “−” denote 0 + (slightly greater than 0 but less than 1) and 10− (greater than 9 but slightly less than 10), respectively. The data sources are indicated in the year line.

For the drift ice amounts, the JMA dataset contains the data during 1956–2003. In addition, the old observation records in *Sea Ice Accumulative-Year Original Ledger* during 1930–1956 are available. In 1956, the daily data from these data sources are partly inconsistent. We considered that some corrections were made for the records for 1956 based on the sketches in digitizing the JMA records. Note that the versions of the ledger agree with each other. For the recent years after 2004, we used the data based on the sketches. Thus, we combined the old ABA data during 1930–1955, the official JMA data during 1956–2003, and the recent ABA data during 2004–2021 and constructed the time series during 1930–2021 as ‘Drift_ice_1930–2021.txt’^25^. The format of the file is the same as the total ice file.

In the dataset^25^, the manually created files (‘SI_dates_1892–2025.txt’, ‘Total_ice_1956–2021.txt’, and ‘Drift_ice_1930–2021.txt’) described above are stored in the directory ‘raw_data’. These data are converted to a more interoperable format for users in the directory ‘csv_data’. The codes for the conversions as well as the figure plots are also shared in the directory ‘codes’. Details of these dataset are described in ‘README.txt’.

## Technical Validation

As the validation of homogeneity for the combined time series of the sea ice dates, we first investigated three standard metrics: level shift, trend shift, and standard deviation (STD) shift from the previous window to the following window. A large value (both positive and negative) at a connection point indicates a caution that the reconstruction of time series might not be robust. Note that natural variabilities and shifts on several time scales are also included, for example, as influenced by the Pacific Decadal Oscillation (PDO)^26^. Thus, the shift values at the connection points are compared with other large values that are known as natural variabilities; if the value at a connection point is as large as or larger than those for the natural shifts, the homogeneity at the connection point is concerned. The window width was set to 15 years (including the target year) to reduce the influence of the decadal variabilities (e.g., PDO). Hence, for the shift metrices in 2000, for example, values for 1986–2000 and 2000–2014 were compared. Level denotes the mean value (day of year) during the window, trend denotes the linear trend, and STD is calculated from differences from the level value.

**Fig. 5:**
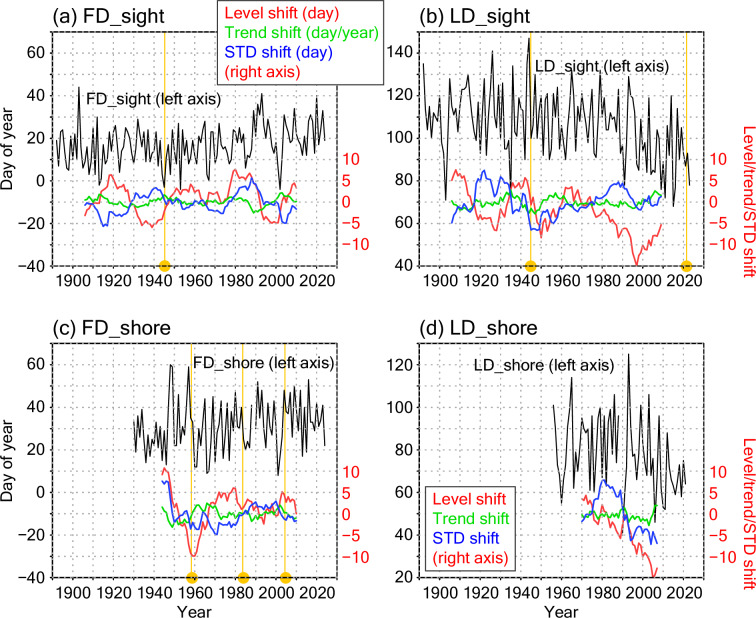
Validation of homogeneity for the sea ice dates. (**a**) FD_sight. (**b**) LD_sight. (**c**) FD_shore. (**d**) LD_shore. Black lines denote the sea ice dates in day of year (accumulative days from 1 January; e.g. 32 for 1 February and −2 for 29 December in the previous year). Validation metrices, level shift (unit in day; right axis), trend shift (day/year), and standard deviation (STD) shift (day) from the previous 15 years to the following 15 years are plotted by red, green, and blue lines, respectively. Since FD_shore and LD_shore were not defined in 1989 because drift ice did not connect to land-fast ice, the metrices were calculated without these data. Data connection years (Table 2) are indicated by yellow vertical lines and circles in (**a–c**).

For the FD_sight time series (Fig. 5a), relatively large level shifts around 1919 and 1939 are perceived before the connection point (1945–1946), but level shifts at similar magnitudes are also seen after that (in the 1980s and the 1990s). Trend shifts are relatively large in magnitude recently with a negative peak around 1989 and a positive peak around 2003. STD shifts are almost within the range from −5 to 5 days. Thus, no peak was detected at the connection point in terms of these validation metrices, supporting the homogeneity of the time series.

For the LD_sight time series (Fig. 5b), the connection points are 1945–1946 and 2021–2022. STD shifts are relatively large (negative) around 1946 but not so larger than other large STD shifts seen in the 1920s and the 1980s; hence, we can consider that artificial shifts do not occur in the former connection point. In terms of the latter connection point, the extended data period of two years is not enough and further validation should be conducted when the data are accumulated in the future, although the time series do not seem unnatural and the final parts of the validation metrices are not so large.

For the FD_shore time series (Fig. 5c), the published JMA records cover the data from 1959 and the data during 1930–1958 was extended in this study. Thus, large (negative) level shifts are seen around the connection point. In addition, relatively large (positive) level and STD shifts are seen around 1945–1946. These variations in level shift seem to result from relatively large FD_shore values (late dates) in the late 1940s and the 1950s. On the large shifts around 1945–1946, although we could not find description of the change in the observation method (definition), changes associated with the JMA establishment might affect the measurements, while there is a possibility that the large shifts reflect the natural decadal variations. We will discuss these shifts in the FD_shore time series later with the drift ice amount data. Other than this possible connection (1945–1946) and the definition changes of FD_shore as described in Method section, changes in the shapes of the breakwaters (as indicated by solid lines toward the lights in Fig. 2b) and in the shadow regions (dashed line in Fig. 2b; e.g. in 2015) due to house building might affect the observation homogeneity near the shore (both FD_shore and LD_shore). Thus, we should note that the homogeneity of the FD_shore time series might be weaker at the connection points than the other sea ice dates. Nevertheless, we retained the extended FD_shore time series since it might reflect an aspect of the local climate and be useful for future studies accompanied by other datasets.

For the LD_shore time series (Fig. 5d), the official JMA data are not extended. Negative level shifts are remarkable after the 1980s similar to LD_sight. These can be physically understood as these sea ice last dates have recently shifted to earlier dates as a result of regional warming^27^. The STD shifts are relatively large around 1980. Again, we could not access whether these STD shifts are detecting artificial connection or not and retained the time series as the original JMA data.

**Fig. 6:**
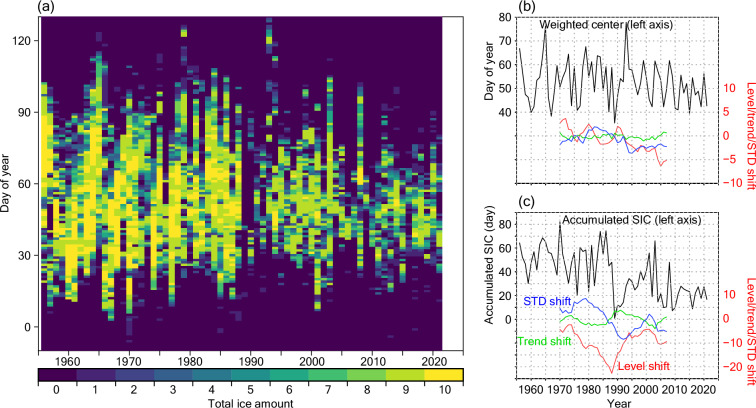
Statistics of total ice amount. (**a**) Daily total ice amount time series in 10th quantile. *x*-axis denotes year and *y*-axis denote day of year. (**b,c**) Weighted center (**b**) and accumulated SIC (**c**) of the total ice for each year (black line). Validation metrices, level shift (unit in day; right axis), trend shift (day/year), and standard deviation (STD) shift (day) from the previous 15 years to the following 15 years are plotted by red, green, and blue lines, respectively.

The daily time series of total sea ice amount (Fig. 6) has already been used in climate studies^11,12^. Although the data in 2021 were added in this study, the data were obtained at the Abashiri Local Meteorological Office with the same manner. In this study, we investigated (1) ice amount-weighted central day (“weighted center”) from the first positive day to the last positive day of the year and (2) accumulated SIC that is sum of the daily SIC values (fraction of 0–1) during the sea ice period, for evaluating the time series. The weighted center time series (Fig. 6b) are mostly within the range 40–70 (day of year), suggesting that the mature stage of total ice generally takes place in February–early March. Although negative level and STD shifts are seen after the 1990s, the magnitudes are much smaller than the metrices for the accumulated SIC (Fig. 6c). In the accumulated SIC time series, remarkable reduction can be seen around 1989, which is reflected in a negative peak of the level shift in the same year and the change of the sign of the trend shift; also the trend shift takes negative values afterward. The above reduction would be related to the abrupt reduction of sea ice over the Sea of Okhotsk in 1989 as described in the previous study^28^.

**Fig. 7:**
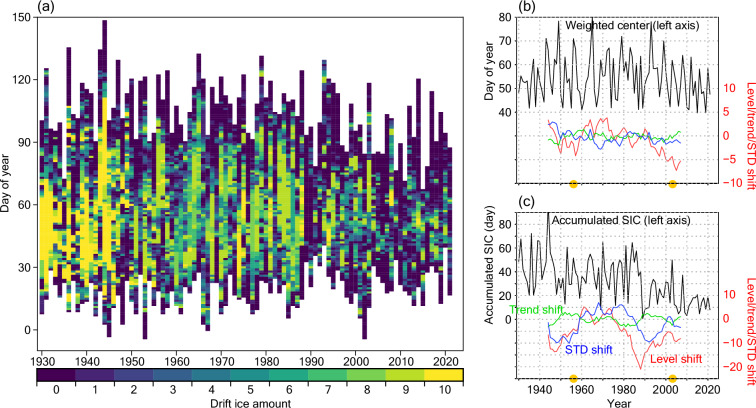
Statistics of drift ice amount. Same as Fig. 6 but for drift ice. For (**a**), only data from FD_sight till LD_sight are plotted for each year. Data connection years (Table 2) are indicated by yellow circles in (**b,c**). Note that drift ice amount values can be zero even on the FD_sight and LD_sight dates since these dates are often determined based on additional observations (other than at 9:00 JST).

For the drift ice amount (Fig. 7), the variations of the validation metrices are similar to those for the total ice amount (Fig. 6); the impacts of the great reduction in 1989^28^ are remarkable in the SIC and the accumulated SIC time series (Fig. 7a,c, respectively). In addition, the longer time series (from 1930) allows us to detect another negative peak of the level shift around the late 1940s. The large accumulated SIC values before the middle of 1940s are consistent with the stably small FD_shore values (early dates) in the same period with small STDs (Fig. 5c). Note that the drift ice amount measurements would be less affected by the near-shore constructions than the FD_shore measurements. Thus, the above consistency lends support to the applicability of the FD_shore time series even in the early years (before 1945). Actually, the previous study^19^ linked the relatively large sea ice amounts in these early years to increasing trend of the surface temperature. This dataset would be of value as a diagnostic tool of the North Pacific climate variability and global warming.

**Fig. 8:**
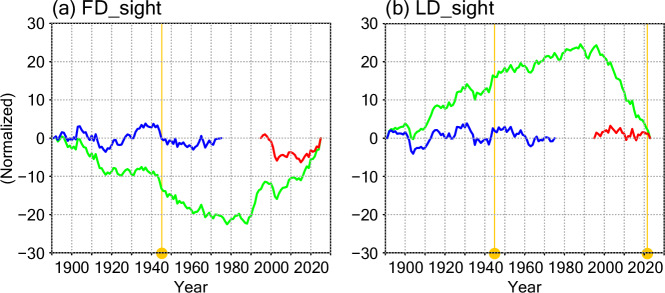
Cumulative deviation from the mean (CDM) time series. (**a**) FD_sight. (**b**) LD_sight. Green lines denote the CDM values for the whole observation periods: 1892–2025 in (**a**) and 1892–2023 in (**b**). Blue and red lines denote the CDM values for the period before 1975 and after 1996, respectively. Data connection years (Table 2) are indicated by yellow vertical lines and circles.

Cumulative deviation from the mean (CDM) approach has also been used for tests of time series homogeneity^29^. We focus on the validation for the relatively long time series, FD_sight and LD_sight. As shown in Fig. 8, the CDM values (green lines; normalized by standard deviation) become as large as 20 for both FD_sight and LD_sight, indicating that the homogeneity is not valid. However, the peak values appear around 1980s, whereas the values at the connection points (vertical yellow lines; Table 2) are relatively small; hence, the discontinuities are attributable to the natural shift as described above^28^. Actually, the CDM peaks are much smaller for the time series before (1892–1975; blue lines) and after (1996–2025; red lines) the discontinuous period due to the natural variation. In these time series (blue and red), the CDM values are rather small in the connection points, suggesting that discontinuities due to the connection of the time series are smaller than the natural variability.

**Table 2:** Connection points of the different data sources to create long time records.

Variable	Connection points
FD_sight	1945–1946
LD_sight	1945–1946, 2021–2022
FD_shore	1958–1959, 1983–1984, 2003–2004
LD_shore	N/A
Total ice amount	N/A
Drift ice amount	1955–1956, 2003–2004

In summary for this validation section, unnatural variations associated with discontinuity of the observation method were not clearly detected. The remarkable variations (i.e. discontinuity) of the total and drift ice amounts in 1989 are consistent with the previous study.

## Data Availability

Codes for technical validation, data format conversion, and figure plots are shared in the ‘codes’ directory in the dataset^25^.
